# Neuron Image Analyzer: Automated and Accurate Extraction of Neuronal Data from Low Quality Images

**DOI:** 10.1038/srep17062

**Published:** 2015-11-23

**Authors:** Kwang-Min Kim, Kilho Son, G. Tayhas R. Palmore

**Affiliations:** 1 School of Engineering, Brown University, Providence, RI 02912, USA; 2 Center for Biomedical Engineering, Brown University, Providence, RI 02912, USA; 3 Department of Chemistry, Brown University, Providence, RI 02912, USA

## Abstract

Image analysis software is an essential tool used in neuroscience and neural engineering to evaluate changes in neuronal structure following extracellular stimuli. Both manual and automated methods in current use are severely inadequate at detecting and quantifying changes in neuronal morphology when the images analyzed have a low signal-to-noise ratio (SNR). This inadequacy derives from the fact that these methods often include data from non-neuronal structures or artifacts by simply tracing pixels with high intensity. In this paper, we describe Neuron Image Analyzer (NIA), a novel algorithm that overcomes these inadequacies by employing Laplacian of Gaussian filter and graphical models (i.e., Hidden Markov Model, Fully Connected Chain Model) to specifically extract relational pixel information corresponding to neuronal structures (i.e., soma, neurite). As such, NIA that is based on vector representation is less likely to detect false signals (i.e., non-neuronal structures) or generate artifact signals (i.e., deformation of original structures) than current image analysis algorithms that are based on raster representation. We demonstrate that NIA enables precise quantification of neuronal processes (e.g., length and orientation of neurites) in low quality images with a significant increase in the accuracy of detecting neuronal changes post-stimulation.

Analyzing morphological changes of a nerve cell (i.e., neuron) is one of the key methods for understanding the behavior of neurons in response to various stimuli (e.g., biochemical, electrical, mechanical, and topographical)^1^,^2^,^3^,^4^,^5^,^6^. Specifically, the length and direction of neurite extension have been used to quantify the effect of a specific cue on neuronal differentiation, neurite outgrowth, and nerve guidance^7^,^8^,^9^. Analyzing images of neurons can be very challenging, however, because neurites are thin (<3.5 μm) arm-like structures and because a high background signal often impedes their accurate visualization. In addition, the length and direction of neurite extension post-stimulation often is determined by manual tracing, a labor-intensive method that can lead to inconsistent results in repeated measurements. To avoid these issues, many image-processing algorithms are being developed that semi-automatically or automatically detect and quantify the morphology of neurons^10^,^11^. These open-source methods use Image J, MATLAB, or Java and have many advantages over commercial software including a reduction in the number of semi-manual annotations required and lower cost. Popular algorithms used to analyze neuronal morphology include skeletonization and edge detection^11^. However, methods that use these algorithms^12^,^13^,^14^,^15^ still require the merging of two separate sets of images to reveal both immnunostained nuclei and neurites in a neural image, and multi-step adjustments to different images (e.g., threshold levels) prior to analysis. More importantly, we will show that using these open-source methods can result in the (1) loss of signal associated with neurites, (2) generation of artifact signals, and (3) false identification of neuronal structures, all of which prevents precise quantification of changes in neuron morphology post-stimulation.

The challenge to analyzing neuron images is mainly due to the low signal-to-noise ratio (SNR) of neuronal structures in images obtained from optical microscopy (e.g., bright field, fluorescent microscopy). Examples of major issues encountered during image processing of neuronal structures are shown in Fig. 1. Current algorithms have difficulty in analyzing the low SNR images because these algorithms do not take into account the relational information of the pixel data. Instead, only pixels with high intensity values determine neuronal structure. Herein we describe Neuron Image Analyzer (NIA), an algorithm designed to harness *relational information between pixels* and thus significantly improve the detection of neuronal structure in images with low SNR. NIA uses the Laplacian of Gaussian (LoG) filter and level set method (LSM) to detect the boundaries of neuronal somata, and employs graphical models (i.e., Hidden Markov Model (HMM) and Fully Connected Chain Model (FCM)) to detect neurites. This study demonstrates that NIA enables specific identification of neuronal structures (i.e., soma, axon, dendrite) and precise quantification of the length and direction of neurites in neuronal images with low SNR.

## Results

### Soma detection

**Figure 2:**
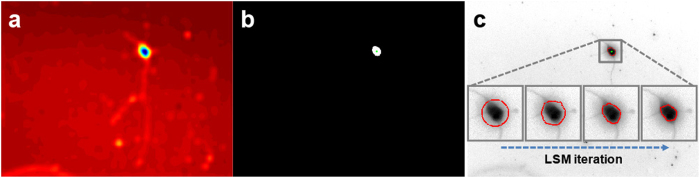
Steps for soma detection. (**a**) *Finding a shape of soma/somata*: the Laplacian of Gaussian (LoG) filter is used to detect blob-shaped structures recognizing that the shape of a soma is approximately an elliptical or circular blob. Blue-to-red color gradient indicates high-to-low response of the LoG filter. (**b**) *Locating the center of a soma*: the filter responses are divided by the maximum response value to generate a response ratio. A threshold is applied to the response ratio to retrieve the soma region (white blob) from the non-soma regions (black). Subsequently, the NMS algorithm is applied to the soma region to locate a local maximum point (green dot), which is used as the center of a soma for neurite tracing. (**c**) *Drawing the boundary of a soma*: LSM detects the boundary of an initial circle whose center was determined by NMS. The diameter of the circle is pre-set at 40 μm, which is slightly larger than that of a soma (15–30 μm). The final shape of the soma is iteratively determined by LSM and serves as the boundary from which neurite tracing begins.

**Figure 3:**
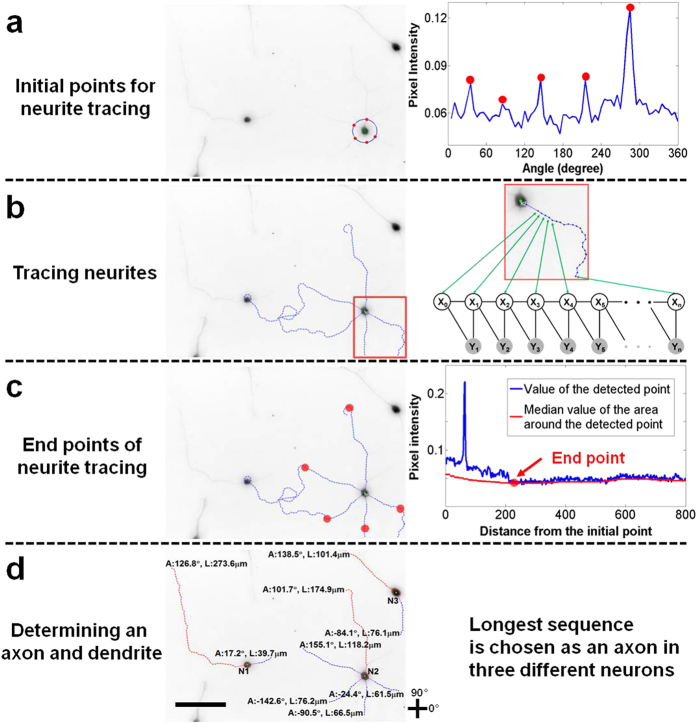
Sequence of steps for detecting neurites. (**a**) *Initial points for neurite tracing*: Neurite detection begins with a circle whose diameter (40 μm) is slightly larger than that of a soma (15–25 μm) and whose center was determined during soma detection. Local maxima on the circle boundary (i.e., signals from neurites) are chosen as the initial points for neurite tracing. (**b**) *Tracing neurites*: Neurite tracing begins at an initial point determined in step (**a**) and is modeled as inferring one-dimensional discrete latent variables 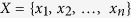 given observations 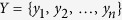 of graphical models (see Fig. 4 for illustration of graphical models). (**c**) *End points of neurites*: Neurite tracing is terminated when the pixel intensity of the detected point is equal to or lower than the local median pixel value of an area (10 × 10 μm) surrounding the detected point. (**d**) *Identifying the axon*: A traced sequence with the longest distance between the initial and end points is identified as the axon (red) with the remaining traced sequences identified as dendrites (blue). Neurite direction is determined from the boundary of the detected soma to the end point of the traced sequence. A soma with a traced sequence shorter than 20 μm or without any traced sequences is not considered a true soma and is removed from the analysis. ‘L’ and ‘A’ represent the length and angle of a neurite, respectively. Scale bar = 100 μm.

Detecting the exact shape of a soma is a pre-requisite to quantifying neurite length and direction. Soma detection based on DAPI staining^12^ or edge detection^14^ generates ambiguous shapes of somata, which results in false identification of a soma and hinders precise measurement of neurite lengths. To address this issue, somata are detected by the Laplacian of Gaussian (LoG) filter as shown in Fig. 2a. The LoG filter generally is used to detect blob-shaped structures^16^, which are similar in shape to a soma (i.e., elliptical or circular). Initially, a threshold to the response ratio (i.e., the filter response value divided by the maximum response value) is applied to soma regions to distinguish them from the background signal (Fig. 2b). Then, the Non-Maximum Suppression (NMS)^17^ algorithm is applied to the soma region to locate a local maximum point (Fig. 2b). This point serves as the center of a soma and is used to detect the soma boundary (Fig. 2c) and to measure neurite length (Fig. 3). The shape of a soma is refined further by applying the Level Set Method (LSM)^18^ to identify the precise location of the soma boundary (Fig. 2c). The boundary of a soma serves as the starting point of neurite tracing as shown in Fig. 3a. In combination with the neurite detection algorithm, a detected soma without a neurite is not considered a true soma and thus ignored in the analysis (supplementary Fig. 1).

### Neurite detection

Neurite detection is an essential part of the process of evaluating neuronal responses to external stimuli. Current neurite detection methods (e.g., edge detection, skeletonization) are based on raster representation, and consequently are prone to generate non-continuous neurites or deform the original structures (e.g., dilation, inconsistent thickness)^12^,^14^. To overcome these inaccuracies, we have modeled neurite detection as a tracing problem, where inferring one-dimensional discrete latent variables are combined with graphical models to give a vector representation of the neurite (Fig. 3). This process begins by finding initial points for neurite tracing. These points are not taken from the boundary of the detected soma because the signal from the soma is far more intense than that from the neurite at that boundary. Instead, the boundary of a circle (40 μm diameter) whose center was determined by NMS is searched for local maxima, and these maxima are used as the initial points for neurite tracing (Fig. 3a). Subsequently, the distance between these initial points and the actual boundary of the detected soma is added to the traced lengths of the neurites to obtain the total length of each neurite:


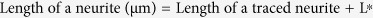


where L* = Euclidian distance between the initial point and the actual boundary of the soma (7.5–12.5 μm)

**Figure 4:**
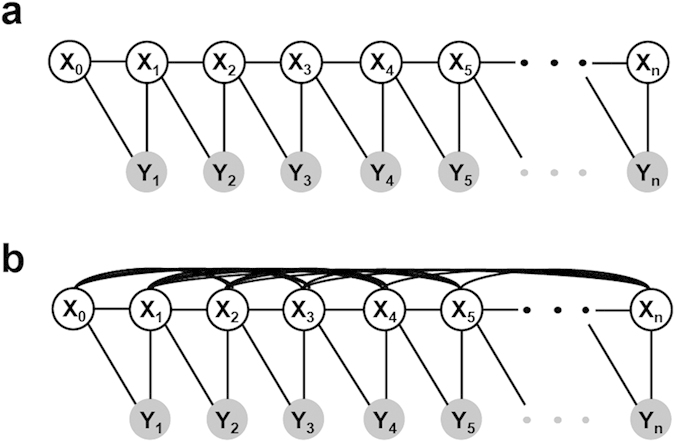
Illustration of graphical models used to detect neurites. The latent variables, 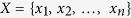, indicate a position of a current tracing point. Their corresponding observations, 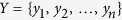, are pixel values between the previous and current tracing points. The links between X-X or X-Y nodes represent pairwise relationships between the variables. The links between X nodes represent geometrical constraints between positions of tracing points whereas the links between X and Y nodes represent the dependency of the observations on the corresponding latent variables. (**a**) Hidden Markov Model (HMM) assumes that the state of the current latent variable (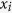) solely depends on the state of the previous latent variable (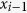) and the observation (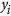) is dependent on the previous and current latent variables (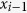,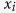). HMM is used widely to analyze structured data by simplifying the relationships between variables (nodes), thereby efficiently and globally inferring the latent variables. **(b**) Fully Connected Chain Model (FCM) assumes that the state of the current latent variable is dependent on all the other latent variables. Unlike HMM, FCM represents all the relationships between latent variables and thus, it is not tractable to globally infer the latent variable. Although it is not suitable for simplifying a given problem, FCM is able to show all the information relevant to the structure-of-interest.

Illustrated in Fig. 4 are the two graphical models (Hidden Markov Model (HMM)^19^ and Fully Connected Chain Model (FCM)^20^) used to trace neurites from the determined initial points. The tracing of a neurite is modeled as inferring one-dimensional discrete latent variables 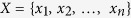 given observations 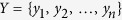 in graphical models (Figs 3b and 4). The latent variables (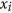) indicate a position of a current tracing point and their corresponding observations (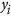) are pixel values determined by the previous and current tracing points of the neurite. HMM assumes that the current state of latent variables depends solely on the state of the immediately previous unknown variable (Fig. 4a and **Methods**). As a result, random detection based solely on pixel intensity is avoided because the traced points are highly interconnected to their adjacent traced points. In addition, the interconnectivity of the traced points enables continuous neurite tracing, where sequential points form a line or path to represent neurite structure without distorting the original structure. Neurite tracing is terminated when the pixel intensity of the detected point is equal to or lower than the median pixel value within an area (10 × 10 μm) surrounding the detected point in the neurite (Fig. 3c). When the number of the traced sequences is two or more, the longest one will be labeled as the axon and the rest as dendrites^21^ (Fig. 3d). Following detection of neurites, NIA measures the length and orientation of the detected neurites. The length of a neurite is calculated based on the scale information of captured images (e.g., 1 pixel = 0.3125 μm). A traced sequence shorter than 20 μm is not counted as a neurite and thus the information is ignored in the analysis. Neurite orientation is determined from the sprouting point on the soma to the end point of the traced neurite.

**Figure 5:**
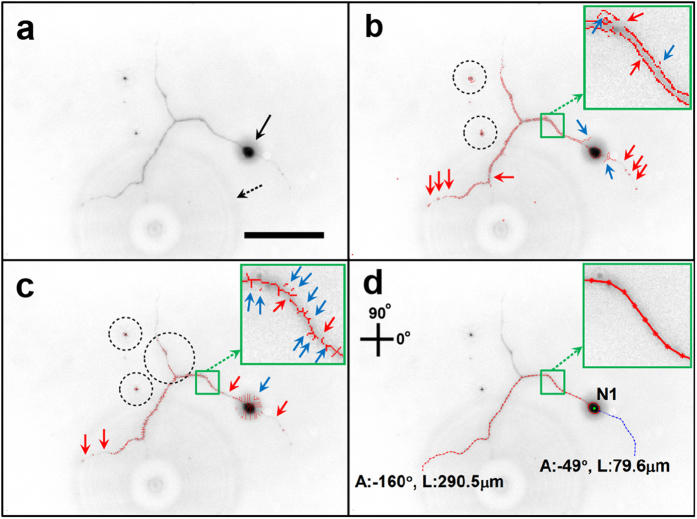
Comparison of neuronal image analysis by different methods. (**a**) Image showing a neuron with a low SNR. The noise includes a small halo around the soma (black arrow) and a large halo in the background (dashed arrow). Scale bar = 100 μm. (**b**) Results from the edge detection method show disconnected neurite structures (red arrows) and spike-like artifact signals around a soma and neurite (blue arrows). In addition, this method generates double stranded structures along the borders of the neurite as shown in the inset, which can multiply the value of neurite length. Non-neuronal particles also are detected (dashed circles). (**c**) Results from the skeletonization method show disconnected neurite structures (red arrows) and spike-like artifact signals (blue arrows). Additional information such as non-neuronal structures or disconnected branches (dashed circles) interferes with analyzing neuronal structures. Thus, the analysis based on raster representation (**b,c**) is likely to prevent precise measurement of neurite morphology. (**d**) Results from NIA method: graphical models used in NIA (i.e, HMM and FCM) enable the analysis based on vector representation where the detected result consists of a sequence of points that are highly relevant to the structure of a neurite. The detected neurite is shown as a single connected line, which prevents the generation of spiky, wavy, and disconnected structures and thus reveals neurite morphology accurately.

Neurite tracing that uses HMM accurately detects neurites compared to those using other image processing methods (Fig. 5). When the neurite has branch nodes, however, HMM has difficulty in identifying the longest neurite (i.e., axon) because this model determines the position of the current point traced based on adjacent positions of tracing points (i.e., Markov property). Consequently, HMM traces a locally brighter neurite at the branch node instead of the longest neurite (see supplementary Fig. 2). Therefore, the Fully Connected Chain Model (FCM) was used (Fig. 4b and **Methods**) to improve the accuracy of detecting the longest length in a branched neurite.

**Figure 6:**
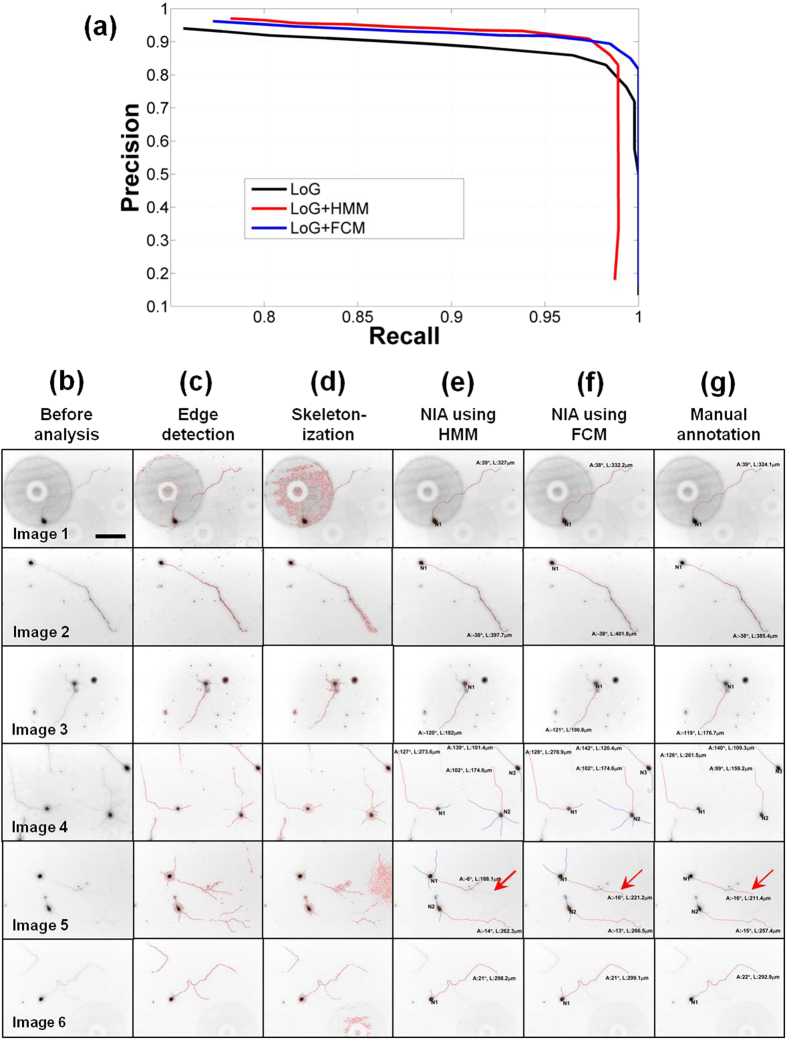
Performance of NIA. (**a**) Precision vs. recall curve of soma detection. The performance of soma detection depends on the threshold parameter of the response ratio (Fig. 2). When the value of the threshold parameter is high, the precision of detection increases and recall is reduced, and vice versa. When soma detection is combined with neurite detection, the precision and recall improve further because somata without neurites are eliminated. LoG (black) represents the precision vs. recall curve using only the soma detector whereas LoG+HMM (red) or LoG+FCM (blue) combine HMM or FCM with the soma detector, respectively. (**b–g**) Side-by-side comparison of various methods used to analyze neuron images. Column (**b**) consists of six images of neurons to be analyzed (Image 1–6). Columns (**c–g**) show the results of image analysis by edge detection, skeletonization, NIA (HMM), NIA (FCM), and manual annotation, respectively. Edge detection (**c**) or skeletonization (**d**) methods falsely identify non-neuronal structures or generate artifacts. In contrast, NIA using HMM (**e**) or FCM (**f**) successfully identifies neuronal structures without deforming the structures or generating artifacts. Furthermore, NIA can identify multi-neurons in a single image (see Images 4 and 5) and when combined with FCM, successfully determines the longest sequence in a branched neurite (red arrows in Image 5). Scale bar = 100 μm.

FCM assumes that the current state is dependent on the states of all the other latent variables (Fig. 4b and **Methods**). NIA using FCM determines the position of the current tracing point from both the adjacent tracing points and all the other tracing points. Thus, when compared to HMM, FCM is more suitable for detecting the longest neurite in a branched neurite because it detects tracing points by exploring the entire structure-of-interest as well as the local one. However, FCM causes Non-deterministic Polynomial time hard (NP-hard) problem where it is not tractable to globally infer the states of the latent variables. To solve this issue, FCM is combined with the variant greedy algorithm that is able to optimally infer the state of latent variables, which is most likely to be the longest branch (Supplementary Fig. 3). Our tests verify that this optimization method (i.e., FCM + variant greedy algorithm) provides physically meaningful results (Fig. 6 and Supplementary Table 1).

### Comparison of NIA with other image processing algorithms

Current image processing algorithms based on raster representation (e.g., edge detection, skeletonization) incorrectly identify particles and debris as neuronal structures, and eliminate or distort data associated with actual neuronal structure, which leads to incomplete detection and inaccurate measurement of neuronal structures (Fig. 5b,c). Thus, these algorithms are not suitable for analyzing neuronal morphologies (i.e., length and orientation of neurites) in images with low SNR. In contrast, the graphical models employed by NIA vector-represent data in images with low SNR to successfully identify neuronal structures (i.e., soma, axon, and dendrite) (Fig. 5d) and accurately measure neurite length and orientation. Our tests verify that NIA generates consistent and reliable results even when the quality of neuron images is varied over a wide range of conditions (e.g., brightness, contrast) (see Supplementary Fig. 4). These tests confirm NIA is robust against different exposure times used during imaging or the type of microscope employed. In addition, NIA significantly improves the accuracy at detecting neurons by combining soma detection algorithms (i.e., LoG, NMS, and LSM) with neurite tracing algorithms (i.e., HMM and FCM) (Fig. 6a). Using this approach, NIA connects only the points geometrically relevant to somata and neurites and thus eliminates the need for multiple sets of immunostained images. Combining the soma and neurite detection algorithms also helps NIA to identify multiple neurons in a single image as shown in the analysis of Image 4 and 5 in Fig. 6. The power of NIA is demonstrated when compared with previous algorithms (Fig. 6b–g).

### Application of NIA to benchmark image sets

Standardized data sets available from the DIgital reconstructions of Axonal and DEndritic Morphology (DIADEM) competition (http://diademchallenge.org) were used to test NIA on more challenging image formats (i.e., stitched or stacked image sets)^22^. Data sets from the DIADEM competition are used to evaluate new methods under development, thus giving researchers a direct comparison of different analytical tools. This type of comparison enables researchers to select the best analytical tool for their specific study.

**Figure 7:**
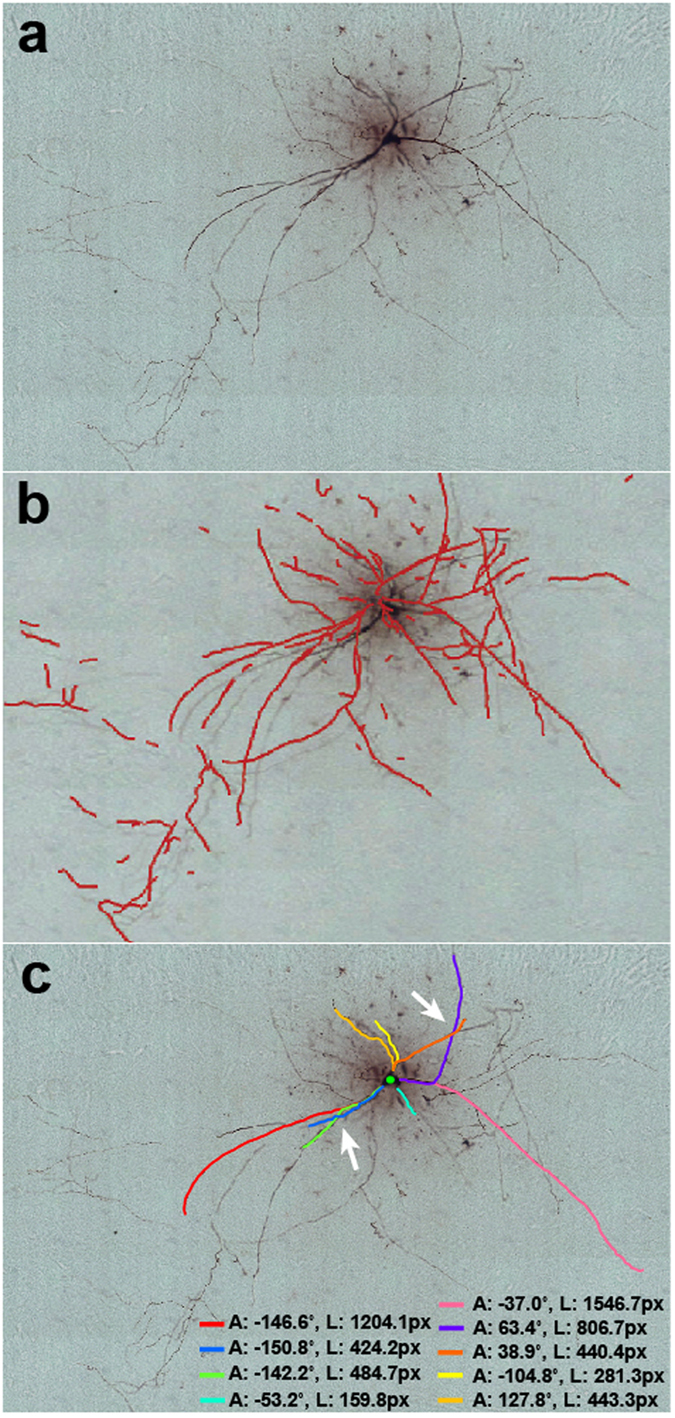
Analysis of stitched images. (**a**) A stitched-image set captured by a transmitted light bright-field microscope shows dendritic and axonal structures of a hippocampal CA3 interneuron^22^. (**b**) Neurite tracing using a neurite fiber graph model that was used in the DIADEM finals competition^23^. This method requires pre-processing for the analysis of bright-field microscopic images and shows numerous discontinuous traces. (**c**) Neurite tracing using NIA. NIA is fully automated without pre-processing of images. NIA traces only the structures extended from soma and identifies crossover neurites (white arrows), which enables NIA to provide individual neurite information. ‘A’ and ‘L’ represent the angle and length of a neurite extension, respectively. The measured values of length are shown in pixels (px) instead of μm because the original image does not include scale information.

For the analysis of stitched images, we tested NIA on an image set^22^ showing a hippocampal CA3 interneuron (Fig. 7). The image set consists of stitched bright-field images (Fig. 7a). In general, stitched images or images generated from simple optical illumination microscopy (i.e., bright-field) have significant changes in the background across stitched images, often requiring additional processing of images prior to analysis to improve detection of neuronal structures^23^. Importantly, NIA does not require pre-processing of images prior to tracing neurites. It is fully automated from image input to quantification of the length and direction of neurites. When compared to the algorithm that was used in the DIADEM final^23^, NIA traces only the neurites that are extended from a soma and excludes information from unidentified or discontinued structures (Fig. 7b,c). In addition, NIA is able to identify neurites that cross over each other (indicated with white arrows in Fig. 7c) because neurite tracing in NIA is based on relational pixel information, not simply on pixel intensity. Thus, NIA is an excellent method for accurate quantification of individual neurites in stitched images.

**Figure 8:**
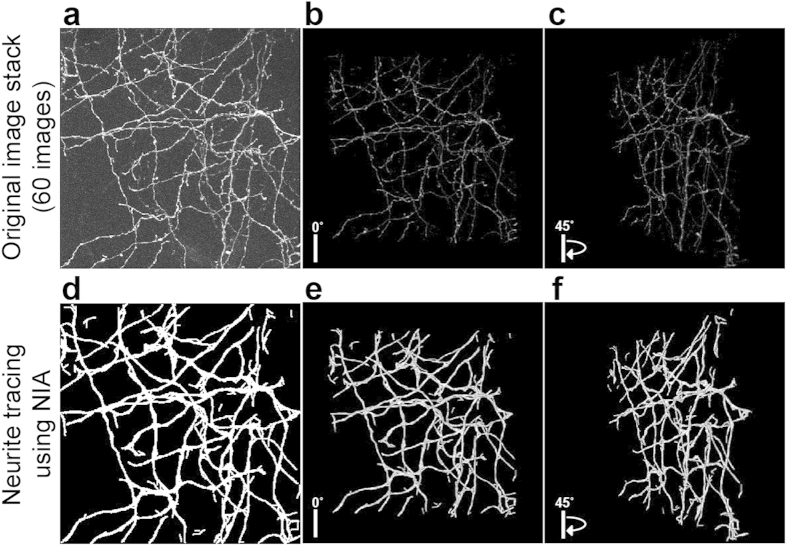
Analysis of stacked images. (**a**) A stack of two-photon laser scanning microscopic images (60 images) shows GFP expressing axons from the mouse neocortical layer 1^22^. Stacked images are shown in 3D projection and rotated with (**b**) 0 and (**c**) 45 degrees. (**d**) Neurite tracing of (**a**) using NIA. Analyzed images are shown in 3D projection and rotated with (**e**) 0 and (**f**) 45 degrees. For better visualization of traced neurites, we have thickened the detected neurite structures. Further comparison of 3D projections is shown in **Videos 1** and **2** (Supporting information). All processes are fully automated.

For the analysis of image stacks, we tested NIA on an image stack^22^ that shows GFP-expressing axons in the mouse neocortical layer 1 (Fig. 8). It is challenging to reconstruct neurite structures in 3D because the light intensity along a neurite is irregular and inconsistent through the image stack and white Gaussian noises exist in the background (Fig. 8a–c). As demonstrated earlier with our results using single images, NIA enables continuous and accurate neurite tracing because it connects only the points that are geometrically relevant to neurite structures. To apply the power of NIA to image stacks, we expanded the analyzing domain from two variables (row, column) to three variables (row, column, depth). We used FCM where each latent node includes three variables (i.e., row, column, and depth) and then, the model was optimized by variant greedy algorithm. When NIA cannot locate a soma that is used as the starting point for neurite tracing, NIA starts tracing from random neurite-like points and later connects the traced sequences. This enables NIA to trace all the neurite structures even when the original image stack does not reveal any soma structure (Fig. 8d). Comparison of 3D projections between the original and the analyzed image stacks demonstrates that NIA successfully traces neurites through stacked images (Fig. 8, Supplementary Videos 1 and 2).

## Discussion

Various algorithms have been employed to improve the manual analysis of neuron images. However, previously developed automated methods have had limited success in detecting and quantifying morphological changes of neurons because these methods simply use pixel intensity values, which are independent, discontinuous, and may not be related to the object of interest. To overcome the limitations of these methods, we developed a new method (NIA) that employs graphical models that detect only geometrically relevant pixels corresponding to neuronal structures. These graphical models (HMM and FCM) enable highly accurate and continuous tracing of neurites. We found that HMM performs better than FCM at analyzing simple neurite structures (i.e., neurite without branches) because the Markov assumption is suitable for modeling simple neurite structures and thus, the objective function of HMM can be optimized globally in polynomial time. In contrast, FCM outperforms HMM at analyzing complicated neurite structures (i.e., neurites with branches) because FCM *excludes* Markov assumptions when analyzing all the information involving neurite structures, which makes FCM more suitable for modeling branched neurites. For comparison, several methods have demonstrated other advanced models (e.g., morphometric statistics, fiber model, Dijkstra algorithm) for tracing neurites with high efficiency and accuracy^23^,^24^,^25^,^26^. These models, however, still require samples to be stained^24^, semi-manual tasks to be performed^24^,^26^, or transmitted light microscopic images to be pre-processed^23^ to complete the analysis of neuronal morphology. In addition, existing image analysis methods are rarely used across different laboratories because the algorithms are not customized to address the variety of biological questions being studied^11^. Thus, manual analysis of neuronal morphology is still the most common approach used. Nevertheless, there remains a strong need for advancing current methods to enable (1) automation without pre-processing, (2) analysis of images captured from low-level light microscopy, and (3) analysis of both single and stitched/stacked images^10^,^11^,^26^. In this regard, NIA is an excellent option because (1) NIA is fully automated and provides quantified information including the length and orientation of traced neurites without any pre-processing or user inputs, (2) NIA demonstrates precise and consistent quantification of neuronal morphology regardless of image quality, and (3) NIA is applicable to not only a single image but also stitched/stacked images that have a variety of background noise. Taken together, NIA will be a useful tool for both beginners and experts who analyze low quality images of neurons obtained via basic optical microscopy as well as high quality images obtained via confocal microscopy. NIA will also be valuable in the analysis of dynamic images captured from live cultures of neurons, where shading and halos often appear due to debris and bubbles in the media.

## Methods

### Primary rat hippocampal neuron culture

Neurons were dissociated from the incubation of hippocampal tissue from E18 Sprague Dawley rat (BrainBits, Springfield, IL) in 2 mL of Hibernate E medium without calcium (BrainBits) containing 4 mg papain (Worthington, Lakewood, NJ) at 30 °C for 30 min. Further dissociation of neurons from tissue was accomplished first by triturating the hippocampal tissue with a fire-polished Pasteur pipette and subsequently transferring the supernatant to a new centrifuge tube. After centrifuging at 1100 rpm for 1 min, the cell pellet was suspended in 2 mL of serum-free culture medium consisting of Neurobasal medium supplemented with 2% B-27 supplement, 0.5 mM L-glutamine (Life Technologies), and 1% antibiotic solution, penicillin (100 U/mL)/streptomycin (100 mg/mL). Cells were seeded on glass coverslips (Fisher Scientific) at a density of 30,000 cells/culture-dish and incubated at 37 °C and 5% CO_2_ under a humidified environment for 3–6 days.

### Immunocytochemistry

All the primary hippocampal cultures were fixed after 3 days *in vitro* (DIV) with 4% paraformaldehyde (Sigma Aldrich) in phosphate buffered saline (PBS, pH 7.4) for 30 min at room temperature, and then rinsed three times with PBS. Fixed samples were permeabilized with 0.25% Triton X-100 (Sigma Aldrich) and then blocked with 10% normal goat serum (NGS, Jackson Immuno Research Laboratories), 2% bovine serum albumin (Sigma Aldrich), and 0.1% Triton X-100 (Sigma Aldrich) in PBS for 1 h at room temperature prior to antibody incubation. Samples were incubated with the primary antibody, a mouse monoclonal antibody RT97 against neurofilament (2 μg/mL, supernatant, Developmental Studies Hybridoma Bank, University of Iowa) overnight at 4 °C. The samples were rinsed three times with PBS and incubated with secondary antibody, Cy2-conjugated goat anti-mouse IgG for neurofilament (1:200, Jackson Immuno Research Laboratories) for 1 h at room temperature. The immunostained samples subsequently were rinsed three times with PBS to prepare for image analysis.

### Microscopy and image analysis

An inverted fluorescent microscope (Nikon Eclipse Ti) was used to image immunostained neurons. Captured images were analyzed by manual annotation, edge detection, skeletonization, or NIA. In the analysis of captured images using manual annotation, imaging software of the microscope (NIS elements, Nikon) was used to examine the length and orientation of neurites. Only neurons that had a neurite longer than 20 μm were chosen for image analysis. The length of axon was measured by manually tracing a neurite from the boundary of the soma to the tip of the axon. The orientation of axon extension was measured as the Euclidian distance from the soma to the tip of the axon. The axon was identified as the longest neurite among those extending from the same soma^21^. The axons connected to adjacent neurons were not measured. For analysis using edge detection and skeletonization methods, morphological functions in MATLAB were applied to neuron images based on previous studies^12^,^14^. For analysis using NIA, captured images were processed with our codes, which implemented with the proposed graphical algorithms (see Figs 2, 3, 4 and **HMM** and **FCM** in **Methods**).

### Hidden Markov Model with dynamic programming

The neurite detection problem is posed as tracing neurite points given the position of an initial tracing point. 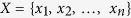 are latent variables where 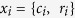. 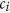 and 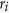 correspond to the column and row position, respectively, of the 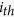 tracing point in the image and 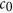 and 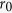 specify the position of the initial tracing point. 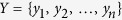 are observation nodes that are pixel intensities on the position determined by the latent variables 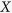. Now, the tracing problem is formulated as inferring discrete latent variables 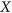 given 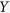 in graphical models (see Fig. 4).

Hidden Markov Model (HMM) assumes Markov properties that the current tracing point solely depends on the very previous tracing point (see Fig. 4a). Latent variables are determined by maximizing a posterior probability (MAP),


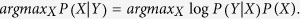


By the Markov assumption,


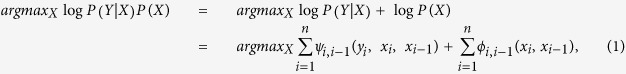


where,


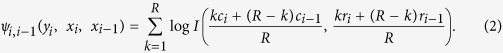


And,


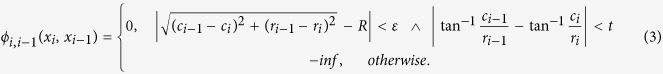


The function 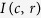 is normalized pixel intensity on the position of column 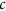 and row 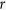 in the input image. The term 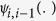 is a log likelihood of tracing points 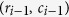 and 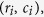 which is determined by latent variables 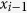 and 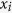. The log likelihood is defined as a sum of log pixel values on the linearly interpolated positions between two tracing points. The prior term 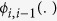 presents geometrical constraints between two adjacent tracing points. The resolution of neurite detection depends on 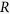 so that the accuracy of neurite length increases by reducing 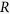 but the computational time increases and vice versa. Here, 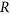 is 15 (pixel), 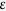 is 0.5 (pixel), and 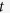
is a possible difference of direction between adjacent tracing points (i.e., the difference of directions between two adjacent tracing points cannot be above 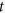.). Dynamic programming globally optimizes the posterior probability with Markov assumption.

### Fully Connected chain Model with variant greedy algorithm

If severe clutter exists in the data (i.e., neurite branching), HMM does not detect the longest neurite because of oversimplification of the problem. In this case, Fully Connected chain Model (FCM) is used, which includes the geometrical constraint that current tracing points are related to all the other tracing points (see Fig. 4b). Latent variables are calculated that maximize a posterior probability,


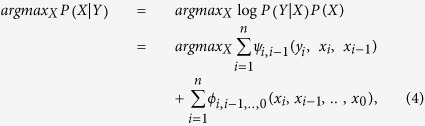


where,


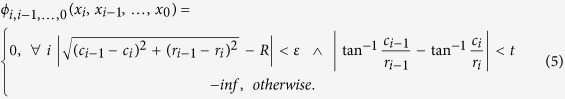


The problem of FCM is that global optimization is intractable in polynomial time because all latent variables are all related to each other. We propose a variant greedy method to optimize the posterior probability efficiently, which is described below.

We start with a single candidate set of tracing points

We initialize the starting point of the tracing candidate with 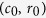


for i = 1:# of latent nodes

for j = 1:# of candidate sets of tracing points

find states of 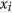
which locally maximizes the equation (2) and satisfies equation (5) = 0

if (exists multiple local maxima) && (# of candidate sets 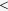 maximum # of candidate sets)

update the i node of j candidate set of tracing points with the local maxima

generate a new candidate set of tracing points with the other local maxima

else

update the i node of j candidate set of tracing points with the local maxima

end

 end

end

find *X* according to equation (4) from the candidate sets of tracing points.

In summary, the tracing points are generated along pixels whose intensity are locally maximum. When the tracing points meet with branches (multiple local maximum of pixel intensity), the algorithm generates a new candidate set of tracing points. Ten sets of candidates are maximumly generated by the method (see Supplementary Fig. 3). The algorithm finds the best set of tracing points, which maximizes the proposed posterior probability, from the 10 sets of candidates.

## Additional Information

**How to cite this article**: Kim, K.-M. *et al.* Neuron Image Analyzer: Automated and Accurate Extraction of Neuronal Data from Low Quality Images. *Sci. Rep.* **5**, 17062; doi: 10.1038/srep17062 (2015).

## Supplementary Material

Supplementary Information

Supplementary Video 1

Supplementary Video 2

## Figures and Tables

**Figure 1:**
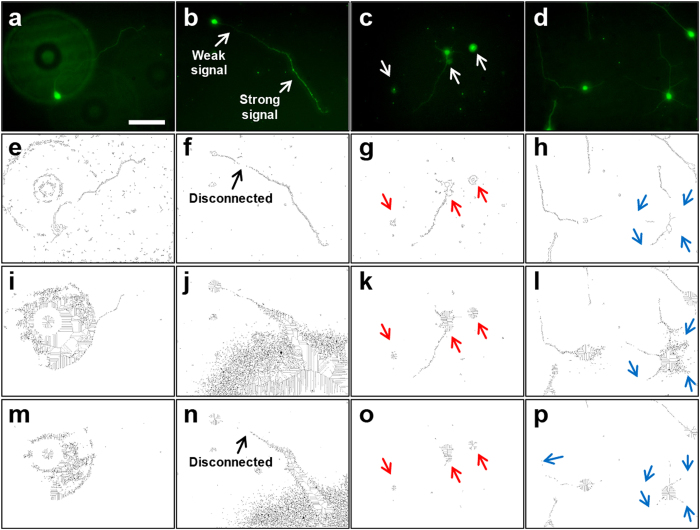
Challenges to analysis of neuronal structures in images with low SNR. Rows of images: (**a–d**) Original fluorescent images of neurons analyzed by various methods. (**e–h**) Edge detection* with manual adjustment of threshold applied to (**a–d**). (**i–l**) Skeletonization applied to (**a–d**) using a low threshold level. (**m–p**) Skeletonization applied to (**a–d**) with a high threshold level to remove additional noise. Columns of images: (**a,e,i,m**) Strong background signal and pixel noise interferes with detection of neuronal structures. Applying a higher threshold to reduce noise (**m**) results in complete loss of neurite. (**b,f,j,n**) Inconsistent signal intensity along a neurite leads to a disconnected neurite. (**c,g,k,o**) False identification of neuronal soma occurs when debris particles are similar in size (indicated by red arrows). (**d,h,l,p**) Neurites lost or overwhelmed by background noise (indicated by blue arrows). Scale bar = 100 μm. *Edge detection in this study used the Canny operator unless specified otherwise.
