# Supplementary material for: Neuron Image Analyzer: Automated and Accurate Extraction of Neuronal Data from Low Quality Images
Source: Sci Rep. 2015 Nov 23;5:17062. doi: 10.1038/srep17062 (PMC4655406; doi:10.1038/srep17062)
Supplement: Supplementary Information [file srep17062-s1.pdf]

Supplementary Information

Neuron Image Analyzer: Automated and Accurate Extraction of Neuronal Data from Low Quality Images

Kwang-Min Kim[1,2,†,‡], Kilho Son[1,‡], and G. Tayhas R. Palmore[1,2,3,*]

[1] School of Engineering, Brown University, Providence, RI 02912, USA

[2] Center for Biomedical Engineering, Brown University, Providence, RI 02912, USA

[3] Department of Chemistry, Brown University, Providence, RI 02912, USA

† Current address: Stanford University School of Medicine, Palo Alto, CA 94304, USA

‡ These authors contributed equally to this work

*Corresponding author: G. Tayhas R. Palmore, 182 Hope Street, Box D, Providence, RI 02912, USA.

Email: tayhas_palmore@brown.edu

Phone: 401 863 2856

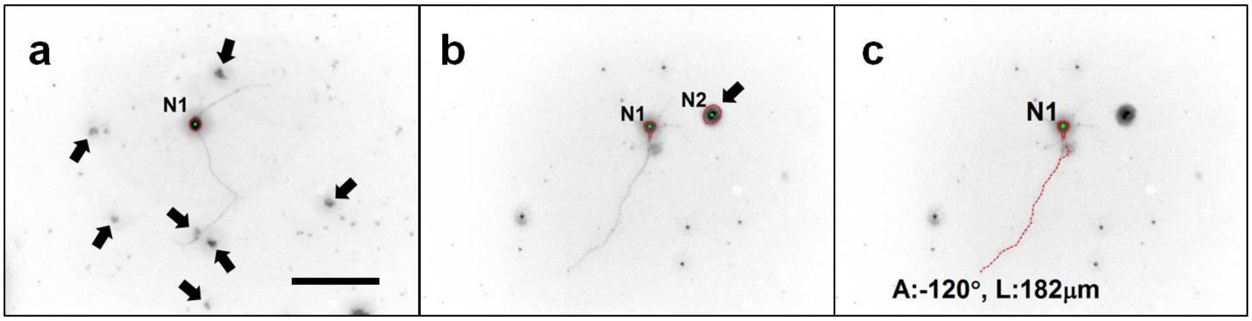

Supplementary Figure 1. Elimination of non-neuronal particles. (a) Shape and size selection of somata: only particles with a blob shape and a size of 15 - 25 μm are selected as soma (e.g., N1) when LoG is used. Black arrows indicate particles that are not blob shaped or are out of range of soma size. Consequently, they are ignored even when geometrical information is present. (b) Possession of a neurite: particles that are blob shaped and 15 - 25 μm in size (e.g., N1, N2) are subjected to the neurite tracing algorithm to determine if neuronal structure is present (i.e., neurite). (c) Particles determined to be without neurites (e.g., N2) are deemed non-soma and removed from further analysis. Scale bar = 100 μm.

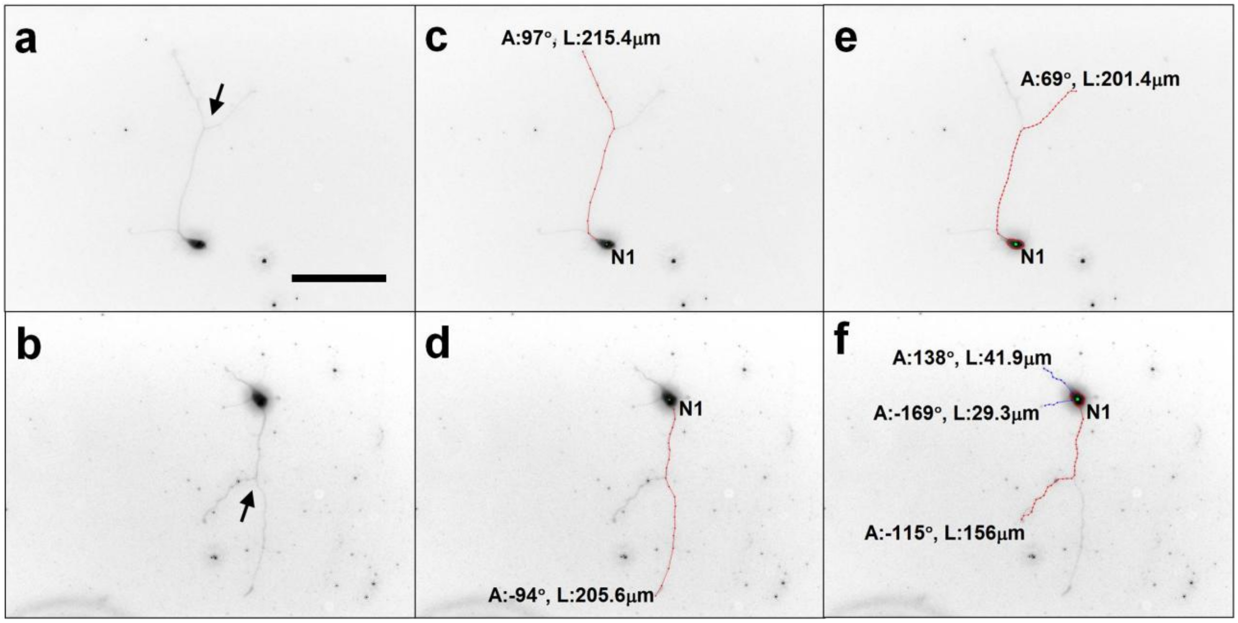

Supplementary Figure 2. Illustration of the limitation of neurite tracing based on HMM. Fluorescent images of neurons with an axon branch node (black arrows) are shown in (a and b) and their corresponding trace as determined by manual tracing (c and d) or HMM (e and f). HMM fails to detect the longest sequence (i.e., axon) when an axon has multiple branch nodes. In multiple tests, HMM detects 78% of axons detected by manual tracing (see **Supplementary Table 1**). Scale bar = 100 μm.

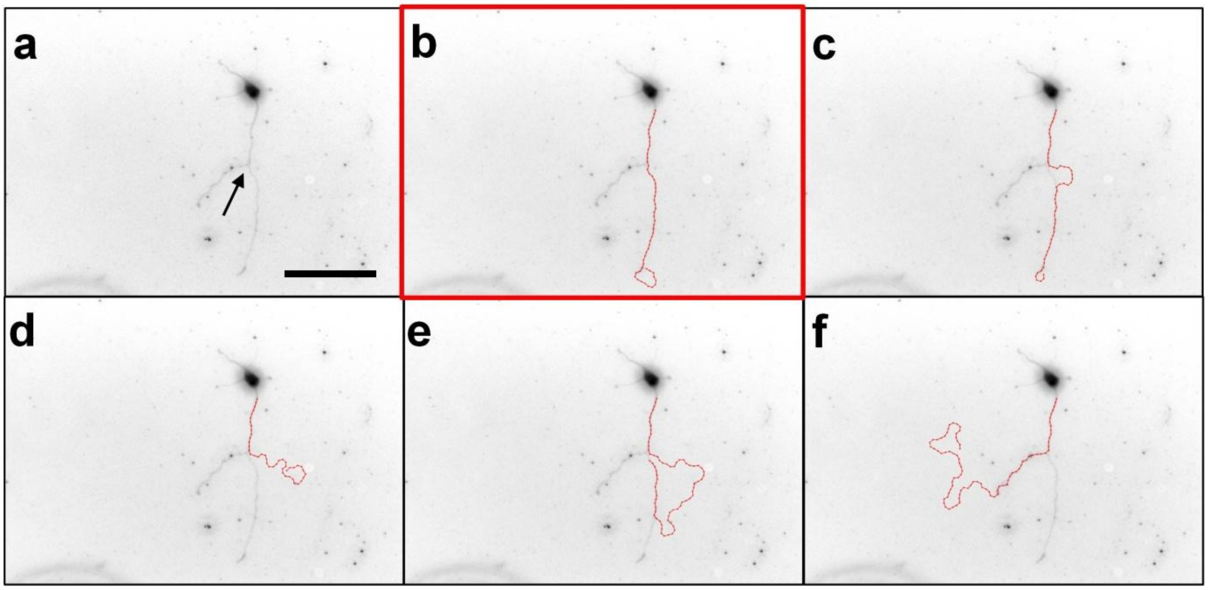

Supplementary Figure 3. Application of the variant greedy algorithm in FCM for the selection of the longest branch: (a) fluorescent image showing a neuron with a branch node (black arrow). (b - f) variant greedy algorithm proposes ten possible neurite branches, five of which are shown here. The neurite shown in (b) is selected because it maximizes the posterior probability (see **Methods**). The scale bar = 100 μm.

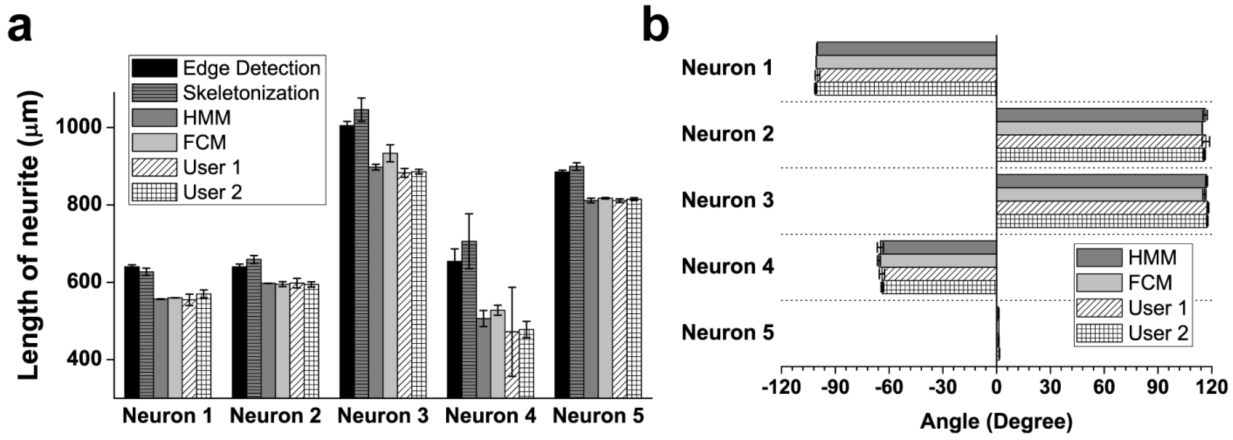

Supplementary Figure 4. Detection accuracy and consistency. Five neuron images are used to test detection accuracy and consistency (Neuron 1 - 5). Each neuron image is shown in ten different conditions (e.g., varying brightness, contrast). (a) The length of the longest neurite under ten different conditions was measured by Edge detection, Skeletonization, NIA (HMM), NIA (FCM), user 1, and user 2. Edge detection and Skeletonization include post-processing of images (e.g., removing large size particles, excluding duplicated tracing). User 1 and user 2 represent manual annotation of soma and neurite by two different researchers. (b) The orientation of the longest neurite under ten different conditions was measured by NIA (HMM), NIA (FCM), user 1, and user 2. We did not include measurement of the orientation of neurites by Edge detection and Skeletonization because Edge detection and Skeletonization generated artifact signals around the end point of the traced neurite, which makes it difficult to determine the direction of neurite outgrowth. The results show that the values measured by NIA are more comparable to those by manual annotations than those by Edge detection and Skeletonization under varied brightness and contrast. More importantly, the values measured by NIA are more consistent than those by manual annotations under varied brightness and contrast. This result suggests that NIA provides reliable and consistent analysis of neuronal morphology regardless of image quality that can be varied by exposure time or equipment type. Error bar = mean ± S.D.

Supplementary Table 1. Success of two models used to detect the longest neurite (456 neurons in the 368 images analyzed) relative to manual tracing.

Manual tracing	HMM	FCM
1.0000	0.7829	0.8581
